# Supplementary material for: A Comparative Analysis of Different Xenorhabdus Strains Reveals a Virulent Factor, Cyclic Pro-Phe, Using a Differential Expression Profile Analysis of Non-Ribosomal Peptide Synthetases
Source: Insects. 2024 Sep 17;15(9):710. doi: 10.3390/insects15090710 (PMC11432276; doi:10.3390/insects15090710)
Supplement: Supplementary file 1 [file insects-15-00710-s001.zip › insects-3196803-SI.pdf]

Supplementary Information

Table S1. Primers used in this study and their PCR conditions

Table S2. Classification of secondary metabolites produced by 6 different *Xenorhabdus* spp.

Fig. S1. 16S rRNA sequencing and BLAST results of Xn-GH and Xn-FK. (A) Xn-GH, (B) Xn-FK.

Fig. S2. Prediction of non-ribosomal peptide synthetase (NRPS) gene localities and domain analysis in the genomes of *Xenorhabdus hominickii*, *Xenorhabdus nematophila*, and *Xenorhabdus bovienii* (GenBank accession number: NZ_CP016176.1, NZ_CP032329, NZ_FO818637). Domain analysis of NRPS genes was performed using the antiSMASH bacterial version software (antismash.secondarymetabolites.org). (A) *X. hominickii*, (B) *X. nematophila*, (C) *X. bovienii*.

Table S1. Primers used in this study and their PCR conditions

Genes	Primers (5’→3’)	Annealing temperature (°C)	Product size (bp)
16S-qPCR-F1	CGACGATCCCTAGCTGGTCTG	55	200
16S-qPCR-R1	ACCGCGGCTGCTGGCACGGAG		
Xh_NRPS1_QPCR_F	CGAAAACAGGTTCCGGCAAG	55	212
Xh_NRPS1_QPCR_R	AACAGGCCGTCGTGATTGAT		
Xh_NRPS2_QPCR_F	ATACAGGCCTTGATGGAAAG	55	165
Xh_NRPS2_QPCR_R	CTGCTTGATGCCTGTTTTAG		
Xh_NRPS3_QPCR_F	ATGAAAGTAACCCAGACACC	55	163
Xh_NRPS3_QPCR_R	CATACTACCCCCTGTCAGTA		
Xh_NRPS4_QPCR_F	GCAGCCACTTTAATGCTAAG	55	195
Xh_NRPS4_QPCR_R	GTTTGGACGATAAGTTGCAG		
Xh_NRPS5_QPCR_F	GTAATAGGCCGATGCAGTAA	53	181
Xh_NRPS5_QPCR_R	CGTGGTACGTCAGATAATGT		
Xh_NRPS6_QPCR_F	GGTTTCGCTGAGGTTAGTAT	53	241
Xh_NRPS6_QPCR_R	GTTCTGTTTCGTCAAATCCC		
Xh_NRPS7_QPCR_F	GTTTACCGGCAGGTTATTTG	55	207
Xh_NRPS7_QPCR_R	CGCCATTACGGAATTGAAAA		
Xh_NRPS8_QPCR_F	CTTGAGCGAACCTGAATTTG	53	172
Xh_NRPS8_QPCR_R	TTAAGCCAGAAAAATCCCGT		
Xn_NRPS1_QPCR_F	CTGGTGGTTTAGAGCTGGCA	55	162
Xn_NRPS1_QPCR_R	AGTAACTCAGCGCGTCAACA		
Xn_NRPS2_QPCR_F	TAAATCCCCGATCCCACCCT	55	349
Xn_NRPS2_QPCR_R	AAACGCAGCCATTGTTGGTC		
Xn_NRPS3_QPCR_F	GGGTGCGACGCATAAATCAC	55	198
Xn_NRPS3_QPCR_R	GTTGATCCGCAACCTCTGGA		
Xn_NRPS4_QPCR_F	TTGAGCCGTTCTCCAATCCC	53	244
Xn_NRPS4_QPCR_R	CGGTATATCCGCAGCTCGTT		
Xn_NRPS5_QPCR_F	CGCTGACGCTGCTGATAAAC	55	336
Xn_NRPS5_QPCR_R	TCGGCACCCAATAAGACACC		
Xn_NRPS6_QPCR_F	AACGTCAGAGGCTTTACGCA	50	140
Xn_NRPS6_QPCR_R	CCCTGAATTGCCTGTTTGCC		
Xn_NRPS7_QPCR_F	GCAACGAAACGGAGTTTGCT	50	262
Xn_NRPS7_QPCR_R	TCATCTCGCGTGATACTGCC		
Xb_NRPS1_QPCR_F	CGCGTGACTTTGGCTTTTGA	55	165
Xb_NRPS1_QPCR_R	CAGCCAGTCATCCACCCATT		
Xb_NRPS2_QPCR_F	CGCGTTATCTTCCGGCTTTG	50	261
Xb_NRPS2_QPCR_R	CCCGGTGTTTGAAATTGCGT		
Xb_NRPS3_QPCR_F	GCAATAAACGGCGCGAGAAT	55	255
Xb_NRPS3_QPCR_R	GGGCAAAACGACAACTTCCC		
Xb_NRPS4_QPCR_F	GGCGACGAATGAAATGGACG	55	224
Xb_NRPS4_QPCR_R	GTCCGGTGCGGATTTTATGC		

Xb_NRPS5_QPCR_F	TACACCAATGGCTCACGCAT	53	350
Xb_NRPS5_QPCR_R	ATAGGGCTGGCAGGTAAGGA		
Xb_NRPS6_QPCR_F	TTCCTCACCGATCCCTTTGC	50	203
Xb_NRPS6_QPCR_R	ACCAGAATCACTGCCTCACG		
Xb_NRPS7_QPCR_F	AGATGCCCGAACTCGGTTTT	55	268
Xb_NRPS7_QPCR_R	CGCTTGATGATTTGGAGGCG		

Table S2. Classification of secondary metabolites produced by 6 different *Xenorhabdus* spp.

Classifications	N	Compounds
Pan. *Xenorhabdus*	34	2,3-Butanediol<br>Butanoic acid, 3-methyl-<br>Pyrazine, 2,5-dimethyl-<br>Nanofin<br>Dimethyl trisulfide<br>Butanamide, 3-methyl-<br>Benzyl alcohol<br>Phenylethyl Alcohol<br>4H-Pyran-4-one, 2,3-dihydro-3,5-dihydroxy-6-methyl-<br>4H-Pyran-4-one, 3,5-dihydroxy-2-methyl-<br>5-Thiazoleethanol, 4-methyl-<br>Indole<br>Phenol, 2,6-dimethoxy-<br>2H-Indol-2-one, 1,3-dihydro-<br>1,3-Dimethyl-3,4,5,6-tetrahydro-2(1H)-pyrimidinone<br>Phenol, 3,5-dimethoxy-<br>7,9-Di-tert-butyl-1-oxaspiro(4,5)deca-6,9-diene-2,8-dione<br>n-Hexadecanoic acid<br>2,5-Piperazinedione, 3-methyl-6-(phenylmethyl)-<br>2,5-Piperazinedione, 3-(phenylmethyl)-<br>2,5-Piperazinedione, 3-benzyl-6-isopropyl-<br>Pyrrolo[1,2-a]pyrazine-1,4-dione, hexahydro-3-(phenylmethyl)-<br>2,5-Piperazinedione, 2-(2-methylthio)ethyl-6-benzyl-<br>D-Tryptophan<br>Methane, sulfomyl tryptophan<br>4-Pentenoic acid, 2-(3-indolylmethyl)-, methyl ester<br>3-Allyl-5-(1H-indol-3-ylmethyl)-2-thioxo-imidazolidin-4-one<br>Dimethyl Sulfoxide<br>2,5-Dimethyl-4-hydroxy-3(2H)-furanone<br>Maltol<br>Pyrimidine, 4,5-diamino-6-methoxy-<br>l-Leucine, N-cyclopropylcarbonyl-, hexadecyl ester<br>9H-Pyrido[3,4-b]indole, 1-methyl-<br>2,5-Piperazinedione, 3,6-bis(phenylmethyl)-
X. nematophila - specific	136	Isoglutamine<br>4-Cyclopentene-1,3-dione<br>Acetamide, N-(aminocarbonyl)-<br>1,2-Cyclopentanedione<br>Carbohydrazide<br>1-Sec-butyldiaziridine<br>Benzaldehyde<br>1,2-Cyclopentanedione, 3-methyl-<br>2-ethyl-1-hexanol,<br>Ethanone, 1-(1H-pyrrol-2-yl)-

		Undecane<br>Undecane, 3-methyl-<br>Cyclopentane, 1-hexyl-3-methyl-<br>2-Ethylhexyl acrylate<br>5-Ethyl-1-nonene<br>3,3,3-Trifluoro-1-propanol<br>Carbonochloridic acid, decyl ester<br>1-Cyano-N-fluoroformimidoyl fluoride (anti)<br>Naphthalene, 2-methyl-<br>Pyrazine, 3,5-dimethyl-2-propyl-<br>Tridecane, 5-methyl-<br>Benzenemethanol, 3-hydroxy-<br>Tridecane, 3-methyl-<br>Biphenyl<br>2-Tetradecene, (E)-<br>Tetradecane<br>Naphthalene, 2,7-dimethyl-<br>Naphthalene, 1,6-dimethyl-<br>Diphenylmethane<br>Naphthalene, 1,8-dimethyl-<br>Cyclopentane, nonyl-<br>Cyclopentane, pentyl-<br>1,1'-Biphenyl, 3-methyl-<br>1,1'-Biphenyl, 4-methyl-<br>Phenol, 2,4-bis(1,1-dimethylethyl)-<br>Naphthalene, 1,4,6-trimethyl-<br>Cyclododecane<br>Pentadecane, 3-methyl-<br>7-Tetradecene<br>Pentadecane, 7-methyl-<br>Pentadecane, 5-methyl-<br>Naphthalene, 1,6,7-trimethyl-<br>Cyclodecane<br>Diethyl Phthalate<br>Cetene<br>7-Hexadecene, (Z)-<br>Hexadecane<br>Methane, di-p-tolyl-<br>Benzaldehyde, 2-hydroxy-3-methoxy-<br>Cyclohexadecane<br>2-Diethylamino-5-methyl-4-oxo-3,4-dihydropyrimidine<br>Tridecane<br>Sulfurous acid, butyl tetradecyl ester<br>Cyclopentane, decyl-<br>2,11-Dodecadiene, 4-acetoxy-<br>Cyclohexanone, 4-hydroxy-4-methyl-<br>(4-Acetylphenyl)phenylmethane

		Heptadecane<br>Heptadecane, 3-methyl-<br>13-Methylnonacosane<br>Tetradecane, 4-ethyl-<br>Ether, dodecyl isopropyl<br>3,3-Diacetyl-2,3,4,5-tetrahydro-2-oxofuran<br>3,4-Dimethoxy-5-hydroxybenzaldehyde<br>Octadecane<br>L-Proline, N-pivaloyl-, ethyl ester<br>1-Octadecene<br>Eicosane<br>Cyclodecane, octyl-<br>Cycloeicosane<br>1,2-Cyclohexanedicarboxylic acid, didecyl ester<br>1-Nonadecene<br>3-Eicosene, (E)-<br>Benzoic acid, 3-(methylthio)-, butyl ester<br>1-Tridecene<br>Nonadecane, 9-methyl-<br>3,3-Diethylheptadecane<br>Heneicosane, 5-methyl-<br>Octadecanoic acid<br>1-Eicosene<br>5-Octadecene, (E)-<br>Captopril<br>Nonadecane<br>5-Eicosene, (E)-<br>1-Tetradecene<br>Pentadecane, 8-heptylidene-<br>Silane, trichlorooctadecyl-<br>Docosane<br>Tetracosane<br>[2-(3,4-Dimethoxyphenyl)ethyl](4-methoxybenzyl)amine<br>Cyclotetracosane<br>Cyclohexane, undecyl-<br>Diethyl 4-methoxybenzylphosphonate<br>3-Amino-4-[4-hydroxyphenyl]butanool<br>Cyclohexane, pentyl-<br>DL-Tryptophan, N-glycyl-<br>Tetrahydro-4H-pyran-4-ol<br>Mepivacaine<br>S-Methyl methanethiosulfinate<br>2(1H)-Pyridinone, 3-methyl-<br>Piperazine, 1-(4-acetylphenylsulfonyl)-4-methyl-<br>Catecholborane<br>N,N'-Nonamethylenebis[-S-3-aminopropyl thiosulfuric acid]<br>Mecysteine

		Naphthalene, 2,3-dimethyl-<br>Pyridine, 2-phenyl-<br>Pentadecane<br>1-(2,2-Dimethylcyclopropyl)-2-phenylacetylene<br>Hexane, 2,3,4-trimethyl-<br>Heneicosane, 11-(1-ethylpropyl)-<br>2-methylhexacosane<br>.gamma.-Guanidinobutyric acid<br>2-Pentylcyclopentanone<br>.gamma.-L-glutamyl-L-glutamic acid<br>Pidolic acid<br>1-(3,3,3-Trifluoro-2-hydroxypropyl)pyrrolidine<br>Bromoacetic acid, 4-methylpentyl ester<br>9,9-Dimethyl-9-silafluorene<br>2(1H)-Naphthalenone, 3,4,4a,5,6,7,8,8a.alpha.-octahydro-5.alpha.-hydroxy-4a.alpha.,7,7-trimethyl-, acetate<br>Cyclotridecanone<br>Furan, 2-methyl-5-(methylthio)-<br>Cyclohexane, 2-propenyl-<br>3,6-Diisopropylpiperazin-2,5-dione<br>Dehydroacetic Acid<br>Cyclohexane, octyl-<br>2,5-Heptadecadione<br>Fumaric acid, butyl hex-4-yn-3-yl ester<br>Heneicosane<br>Z-11-Tetradecenoic acid<br>8-Heptadecene<br>Phthalic acid, di(2-propylpentyl) ester<br>.gamma.-Gurjunenepoxide-(2)<br>9-Hexacosene<br>1-Docosene<br>DL-Tryptophan<br>1,2-Bis(trimethylsilyl)benzene
X. hominickii -specific	88	1,2-Propanediol diformate<br>Piperidine, 2-propyl-, (S)-<br>N-(4-Iodo-2-methyl-phenyl)-2-piperidin-1-yl-acetamide<br>2-Cyclopenten-1-one, 2-hydroxy-3-methyl-<br>2-Pyrrolidinone<br>Succinimide<br>Phenol, 3-amino-<br>3-Pyridinol, 2,6-dimethyl-<br>3,4-dimethyl-1H-pyrrole-2-carboxaldehyde<br>2-Piperidinecarboxylic acid, tert-butyldimethylsilyl ester, (DL)-<br>1,2-Dimethylbenzimidazole<br>Niacinamide<br>1H-Benzotriazole, 5-methyl-<br>Ethyl 4-t-butylbenzoate

		2-Aminocycloheptaimidazol-6-ol<br>Quinoline, 7-methyl-<br>2-Thiazoleamine, 4-(tetrahydro-2-oxofuran-5-yl)-<br>3-Morpholinopropyl 2,4-dihydroxybenzoate<br>3,5-Dimethoxytoluene<br>4-Trimethylsilyloxyaniline<br>2-Ethylpiperidine<br>3,5,7,8-Tetrahydro-4,6-pteridinedione<br>Benzenamine, 2-methoxy-N-(3-pyridinylmethylene)-<br>Propionamide, N-(6-fluorobenzothiazol-2-yl)-<br>3,7-Diazabicyclo[3.3.1]nonane, 9,9-dimethyl-<br>Hexadecane, 7,9-dimethyl-<br>Bicyclo[2.2.1]heptane-1-carboxylic acid, 7,7-dimethyl-<br>1-Heptadecene<br>3-Methoxy-4-nitrobenzyl alcohol, methyl ether<br>1-Cyclohexyl-5-(4-methoxy-benzyl)-pyrimidine-2,4,6(1H,3H,5H)-trione<br>2-(3,3-Dimethyl-but-1-ynyl)-2,6,6-trimethyl-cyclohexane-1,4-dione<br>4H-[1,2,4]Triazole, 3-(4-methoxybenzylsulfanyl)-4-methyl-<br>Indolebutyric acid<br>2-Butanol, 3-methyl-<br>2,3-Butanediol, [R-(R*,R*)]-<br>Silane, dimethyl-<br>Serine<br>3-Thietanol<br>Phenol<br>4-Heptanol, 2,6-dimethyl-<br>Pantolactone<br>3-Oxobutan-2-yl 2-methylbutanoate<br>Hexanoic acid, 4-methyl-<br>N-Hydroxymethylacetamide<br>4-Pyridinecarboxylic acid, methyl ester<br>5-Methyl-2-pyrazinylmethanol<br>Isovaleric acid, 4-methoxy-2-methylbutyl ester<br>Dehydromevalonic lactone<br>Benzoic acid<br>N-Aminopyrrolidine<br>2-Coumaranone<br>dl-Mevalonic acid lactone<br>Indolizine<br>2-Methoxy-4-vinylphenol<br>1,2,4-Trimethoxybenzene<br>DL-Proline, 5-oxo-, methyl ester<br>Benzeneethanol, alpha.-(phenylmethyl)-<br>Benzeneethanol, alpha.-methyl-<br>2,6-Dimethylphenyl isocyanate

		Acetamide, N-(2-phenylethyl)-<br>Thiourea, ethyl-<br>10-Methylundecan-5-olide<br>2,5-Diethoxy-3-methyl-2,5-dihydro-furan<br>4,4'-Dimethylbiphenyl<br>6-(2-Hydroxyethyl)amino-3-ethylthio-1,2,4-triazolo[4,3-b][1,2,4,5]tetrazine<br>Hexadecanamide<br>endo-3-Methylenetricyclo[3.2.1.0(2,4)]oct-6-ene<br>Benzoic acid, 2,4-bis[(trimethylsilyl)oxy]-, trimethylsilyl ester<br>(2,3-Difluoro-5-methyl-phenyl)-pyrrolidin-1-yl-methanone<br>1-Aza-2-sila-5-boracyclopent-3-ene, 5-(1,1-dimethylethyl)-4-ethyl-1,2,2,3-tetramethyl-<br>8-Hexadecenal, 14-methyl-, (Z)-<br>Pyrrolo[1',2':1,2]imidazo[4,5-e][1,4]diazepine-2,5(1H,7H)-dione, 3,4,8,9-tetrahydro-<br>o-Butyl O,O-diethyl phosphorothioate<br>3H-Benzo[4,5]imidazo[2,1-c][1,2,4]triazole, 3-tert-butyl-3-methyl-2,9-dihydro-<br>6-Methoxytryptoline<br>Propanamide, 2-amino-3-(3-indolyl)-<br>Cyclononasiloxane, octadecamethyl-<br>1,16-Hexadecanediol<br>3-Benzylidene-hexahydro-pyrrolo[1,2-a]pyrazin-1,4-dione<br>3,4-Dimethoxycinnamic acid<br>3-Pyridinecarbonitrile, 2-(1,3-benzodioxol-5-yloxy)-<br>Bibenzyl, 4,4'-dimethoxy-<br>Benzenemethanol, .alpha.-(chloromethyl)-<br>4-(2,4-Dimethyl-phenylcarbamoyl)-butyric acid<br>Cyclo-(l-leucyl-l-tyrosyl)<br>Ethyl N(alpha)-acetyl-dl-tryptophanate<br>Silane, trimethyl[5-methyl-2-(1-methylethyl)phenoxy]-<br>1,4-Bis(trimethylsilyl)benzene
X. bovienii -specific	65	R-(-)-1,2-propanediol<br>Silane, ethyldimethyl-<br>(E)-3-(Dimethylamino)-2-pentene<br>Ethanol, 2,2'-dithiobis-<br>1,1-Bis[aziridyltrimethylamine]<br>Benzen-d5-amine<br>1,3-Dioxol-2-one,4,5-dimethyl-<br>2,2-Dimethyl-3(2H)-furanone<br>Pyrazine, tetramethyl-<br>Cyclobutene, 2-propenylidene-<br>Cyclohexanone, 4-ethoxy-<br>2,3,5-Trimethyl-6-ethylpyrazine<br>2-Methyl-3-methoxy-4H-pyran-4-one

		3-Fluoro-N-[2-(N-hydroxycarbamimidoyl)-ethyl]-N-methyl-benzamide<br>n-Decanoic acid<br>N,N-dimethyl-P,P-bis(1-methylethyl)-phosphinous amide,<br>4-amino-6-(ethylamino)-1,3,5-triazin-2(1H)-one<br>2,4,6-Trimethyl-1,3-phenylenediamine<br>N-Butyryl-DL-homoserine lactone<br>cis-5-Dodecenoic acid<br>1-Naphthalenemethanamine<br>2-(Hexamethyleneimino)ethanol<br>N-butyl-N-2-propenyl-1-butanamine<br>octahydro-1-benzoxepin-2(3H)-one<br>4-Mercaptophenol<br>2H-Imidazole-2-thione, 1,3-dihydro-1-methyl-<br>2-Piperidinone, 1-(3,4,5,6-tetrahydro-2-pyridinyl)-<br>2-(1-methylethyl)-naphthalene<br>O,O'-Bis(2-diisopropylaminoethyl) methylphosphonite<br>(E)-9-octadecenoic acid<br>1-Nitro-2-pentyloxybenzene<br>6-Tridecene<br>(1S,9aS,Z)-3-((2R)-5-Hydroxy-2-methylhexylidene)-1-methyloctahydro-1H-quinolizin-1-ol<br>1-chloro-7-heptadecene<br>2,3,4,5-tetramethyl-1,4-hexadiene<br>2-(4-Hydroxy-6-methylpyrimidin-2-ylsulfanyl)-N-(4methylthiazol-2-yl)acetamide<br>1-methoxy-4-undecyl-benzene<br>methyl ester-L-tryptophan<br>N-formyl-l-tryptophan<br>5-Chloro-1-indanone<br>2-ethoxy-ethanol<br>2-(2-(2-(2-(2-Methoxyethoxy)ethoxy)ethoxy)ethoxy)acetic acid<br>4H-Pyran-4-one, 3-hydroxy-2,6-dimethyl-<br>azido-benzene<br>2,5-Dimethyl-2,4-dihydroxy-3(2H)-thiophenone<br>1-Methyl-1,6-diazaplenalene<br>trans-Cinnamic acid<br>1-Methyl-2-butyl-5-heptylpyrrolidine<br>2,5-bis(1,1-dimethylethyl)-phenol<br>Uracil<br>2-Acetoxy-5-(2-chloroethyl)-4-methylthiazol<br>3,4-dimethoxy-phenol,<br>Pelletierine<br>4-Fluorophenylhydrazine<br>N-Hexanoyl-DL-homoserine lactone<br>2,4,5-Trihydroxypyrimidine<br>N-glycyl-DL-threonine

		d-Proline, N-allyloxycarbonyl-, heptadecyl ester<br>1,2-Cyclohexanedicarboxylic acid, diisopropyl ester<br>Glutarimide, N-dodecyl-<br>Benzenemethanol, .alpha.-(2-aminocyclopentyl)-<br>(1α,2α.,3β)-Dimethyl-1,3-dimethyl-4-cyclohexene-1,2-dicarboxylate<br>6-[4-Methoxybenzyloxy]-8-nitrolepidine<br>1-(4-Hydroxy-phenyl)-2-(1-p-tolyl-1H-tetrazol-5-ylsulfanyl)-ethanone<br>(3S,5R,8aR)-3-(Hex-5-en-1-yl)-5-propyloctahydroindolizine

(A)

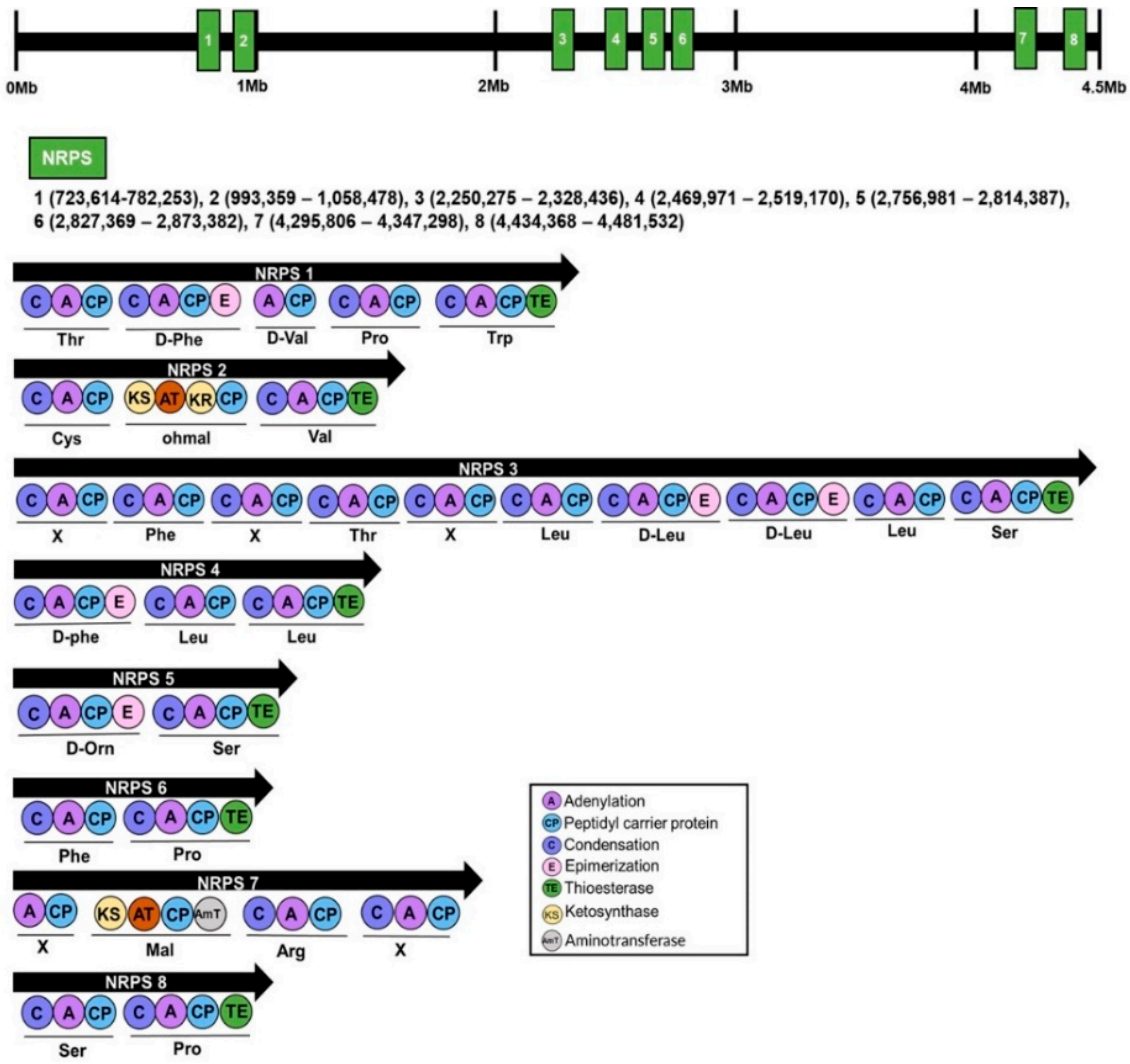
0Mb
1Mb
2Mb
3Mb
4Mb
4.5Mb
NRPS
1 (723,614-782,253), 2 (993,359 – 1,058,478), 3 (2,250,275 – 2,328,436), 4 (2,469,971 – 2,519,170), 5 (2,756,981 – 2,814,387), 6 (2,827,369 – 2,873,382), 7 (4,295,806 – 4,347,298), 8 (4,434,368 – 4,481,532)
NRPS 1
Thr
D-Phe
D-Val
Pro
Trp
NRPS 2
Cys
ohmal
Val
NRPS 3
X
Phe
X
Thr
X
Leu
D-Leu
D-Leu
Leu
Ser
NRPS 4
D-phe
Leu
Leu
NRPS 5
D-Orn
Ser
NRPS 6
Phe
Pro
NRPS 7
X
Mal
Arg
X
NRPS 8
Ser
Pro
Adenylation
Peptidyl carrier protein
Condensation
Epimerization
Thioesterase
Ketosynthase
Aminotransferase

(B)

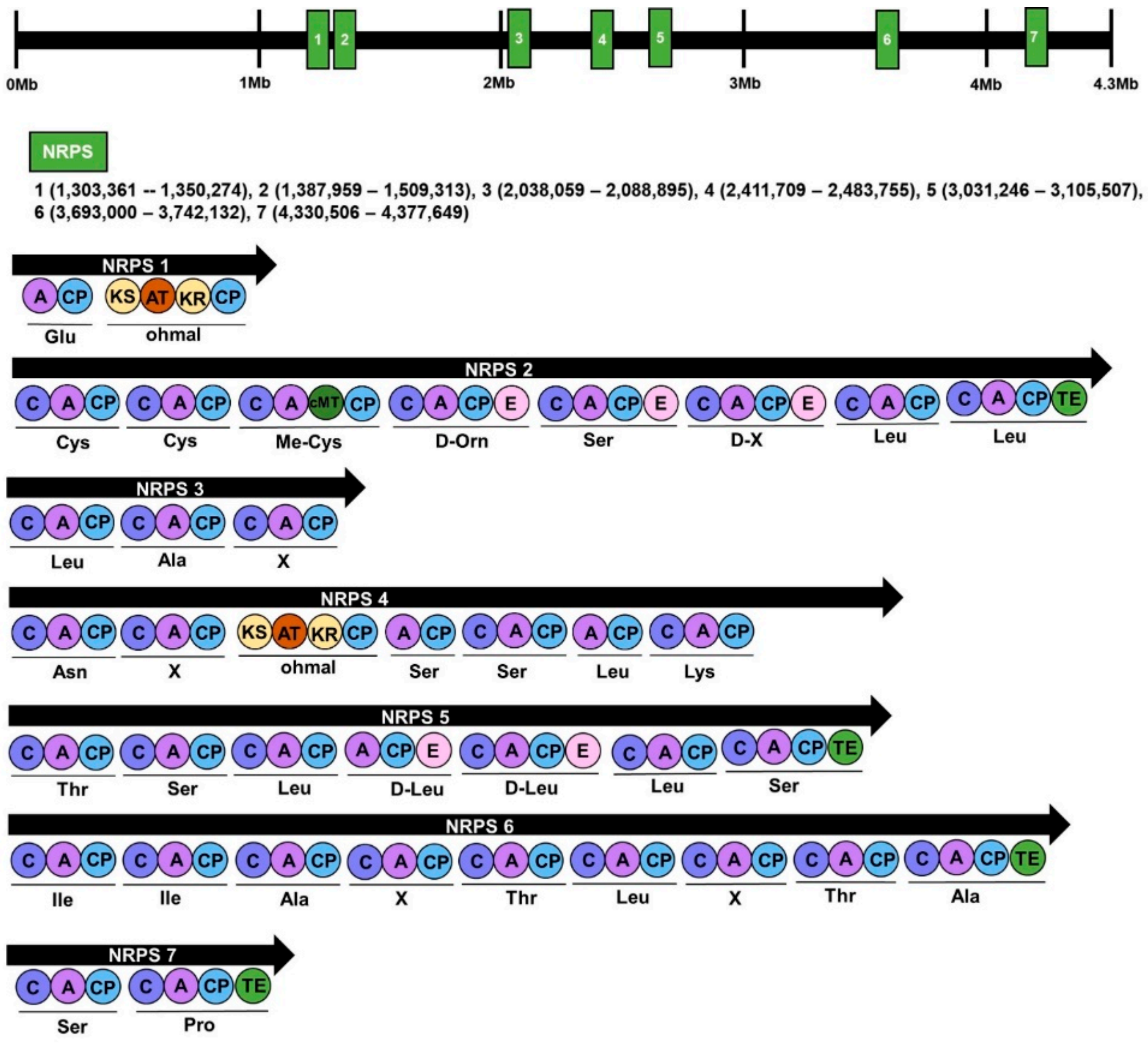
0Mb
1Mb
2Mb
3Mb
4Mb
4.3Mb
NRPS
1 (1,303,361 -- 1,350,274), 2 (1,387,959 – 1,509,313), 3 (2,038,059 – 2,088,895), 4 (2,411,709 – 2,483,755), 5 (3,031,246 – 3,105,507), 6 (3,693,000 – 3,742,132), 7 (4,330,506 – 4,377,649)
NRPS 1
Glu
ohmal
NRPS 2
Cys
Cys
Me-Cys
D-Orn
Ser
D-X
Leu
Leu
NRPS 3
Leu
Ala
X
NRPS 4
Asn
X
ohmal
Ser
Ser
Leu
Lys
NRPS 5
Thr
Ser
Leu
D-Leu
D-Leu
Leu
Ser
NRPS 6
Ile
Ile
Ala
X
Thr
Leu
X
Thr
Ala
NRPS 7
Ser
Pro

(C)

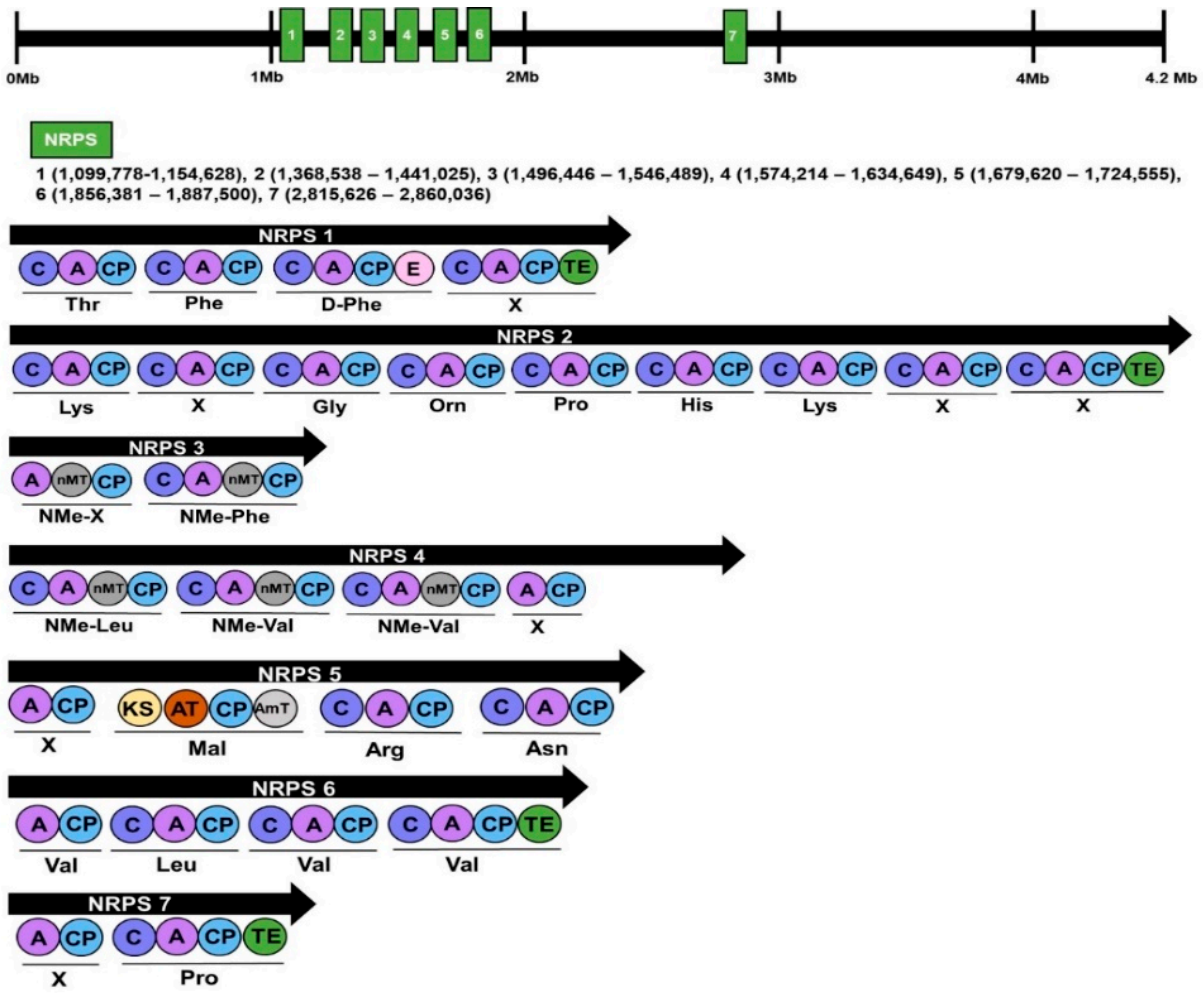

1
2
3
4
5
6
7
0Mb
1Mb
2Mb
3Mb
4Mb
4.2 Mb
NRPS
1 (1,099,778-1,154,628), 2 (1,368,538 – 1,441,025), 3 (1,496,446 – 1,546,489), 4 (1,574,214 – 1,634,649), 5 (1,679,620 – 1,724,555),
6 (1,856,381 – 1,887,500), 7 (2,815,626 – 2,860,036)
NRPS 1
C A CP C A CP C A CP E C A CP TE
Thr
Phe
D-Phe
X
NRPS 2
C A CP C A CP C A CP C A CP C A CP C A CP C A CP C A CP C A CP TE
Lys
X
Gly
Orn
Pro
His
Lys
X
X
NRPS 3
A nMT CP C A nMT CP
NMe-X
NMe-Phe
NRPS 4
C A nMT CP C A nMT CP C A nMT CP A CP
NMe-Leu
NMe-Val
NMe-Val
X
NRPS 5
A CP KS AT CP AmT C A CP C A CP
X
Mal
Arg
Asn
NRPS 6
A CP C A CP C A CP C A CP TE
Val
Leu
Val
Val
NRPS 7
A CP C A CP TE
X
Pro

Figure S1

(A)

TCTGCCCGATGGAGGGGGATAACCACTGGAAACGGTGGCTAATACCGCATGACCTCTTGGGAGTAAAGTGGGGGA
CCTTCGGGCCTCACGCCATCGGATGAACCCAGATGGGATTAGCTGGTAGGCGGGGTAATGGCCCACCTAGGCGAC
GATCCCTAGCTGGTCTGAGAGGATGACCAGCCACACTGGGACTGAGACACGGCCCACACTCCTACGGGAGGCAGC
AGTGGGGAATATTGCACAATGCCCGCAAGCCTGATGCAGCCATGCCGCGTGTATGAAGAGGGCCTTCGGGTTGTA
AAGTCCTTTCATCTGGTAGGGGGGGGGAAATCTGAGGGGTGCTTACTATTAATGATGCCCGCGGATATTTCCCCC
CCCCCTCCCTACTGGTGGAAGCACAAAAAAAAATATAGCAAGCGTGGTGCGGAGACTGCACCATAATGTATATGA
TCGTGGCCAGCTAACGTGACGGGGTGATGGGAGTTTTATCGAGTAGAAAAAATCTTACCGTAGCTTCTAGGATCC
CGGCTGCGGCGGCGGATAAAGAATGTGAGGCTACAGCCTGTTTTTGGGTTGGAACAGGACGGGAACTACCGGGGT
TACCTAACCCCTGTTTCGCCCCCACTCTTGCCGCCGGAAGCGGGGAGTCTTCCGTCCGAGGGGCCGCCTTCCGCA
GCGGTGTTTCCGCCACACCTTAAGAAATTCCCCGGCTGACGCGGGGAAATCCCCCCCCCCCCCCAAAATTTCCTT
CCCCTACCTCCGGTGGAAGCCGTTCCGGGGTTAAACCCGGAGGATTCCACTCCCCGTGTAATTCGACCGCCGGGA
TGGCTTTTCCCCCCAAGATTCCAGGGAACCCTGGCCCCCCGCGATGTACCCGGGCGGGGAGGGAAGGGAGATTA
GCCCGGGCTTTTCTTGCGCGGAGAAACGATCAAAGAAAAACCGCTGTTAAAAAGTAGACGCTTTCCCCCCCGAGG
AAAGGGTGTGTTCGCGCCACGGAATGCCCCTTCTTTCGCGCCCGGGGGGGGGGGTTAGCCACGGCGGGAGGGCCC
CAGTAAGGGAAAAAATCCCCCCCCTGCGGCCCACCACCCAGAAGGGAGGG

Description	Max score	Total score	E-value	Per.ident (%)
Xenorhabdus nematophila strain NN9	547	547	2e-150	99.39
Xenorhabdus nematophila strain SK1	547	547	2e-150	99.39
Xenorhabdus nematophila strain YL001	547	3789	2e-150	99.39
Xenorhabdus nematophila strain USGA01	547	547	2e-150	99.39

(B)

TGGCAATGCGGGAGGTTAACACATGCAAGTCGTGACGGTGAGTCAGCCAGATAAGCTTGCTGTGTTTTGCTGACG
AGTGGCGGACGGGTGAGTAATGTCTGGGGATCTGCCCGATGGAGGGGGATAACCACTGGAAACGGTGGCTAATAC
CGCATGACCTCTTGGGAGTAAAGTGGGGGACCTTCGGGCCTCACGCCATCGGATGAACCCAGATGGGATTAGCTG
GTAGGCGGGGTAATGGCCCACCTAGGCGACGATCCCTAGCTGGTCTGAGAGGATGACCAGCCACACTGGGACTGA
GACACGGCCCAGACTCCTACGGGAGGCAGCAGTGGGGAATATTGCACAATGGGCGCAAGCCTGATGCAGCCATGC
CGCGTGTATGAAGAAGGCCTTCGGGTTGTAAAGTACTTTCAGCGGGGAGGAAGGCGTAAGTCTGAACAGGGCTTA
CGATTGACGTTACCCGCAGAAGAAGCACCGGCTAACTCCGTGCCAGCAGCCGCGGTAATACGGAGGGTGCAAGCG
TTAATCGGAATTACTGGGCGTAAAGCGCACGCAGGCGGTCAATTAAGTTGGATGTGAAATCCCCGGGCTTAACCC
GGGAACGGCATCCAAGACTGGTTGGCTAGAGTCTCGTAGAGGGGGGTAGAATTCCACGTGTAGCGGTGAAATGCG
TAGAGATGTGGAGGAATACCGGTGGCGAAGGCGGCCCCCCTGGAACGAAGACTGACGCTCAGGTGCGAAAAGCGT
GGGGAGCAAACAGGATTAGATACCCTGGTAGTCCACGCTGTAAACGATGTCGATTTGGAGGCTGTGCCCTTTGAG
GCGTGGCTTCCGGGAGCTAACGCGTTAAAATCGACCGCCTGGGGGAGTACGGCCCGCAAGGTTAAAACTCAAATG
AATTGACGGGGGCCCGCACAAGCGGTGGAGCATGTGGTTTAAATTCGATGCAACGCGAAGAAACCTTACCTACTC
CTTGACATCCACGGGAATCAGGCAGAAAATGCCGGAAATGCCTCTCGGGAAAACCCGGGAAACAAAGGGCCTCCC
TGGCCTTTCCTCCCCCCCCCCGGGTTTGGAAAAAAATTTGGGGTTTAAAAACCCCCCCAAAGAGGGCCAAA

Description	Max score	Total score	E-value	Per. ident (%)
Xenorhabdus nematophila strain Xn	1698	1698	0.0	97.42
Xenorhabdus nematophila strain ATCC 19061	1690	1690	0.0	96.96
Xenorhabdus nematophila strain K102	1690	1690	0.0	96.96
Xenorhabdus nematophila strain K97	1690	1690	0.0	96.96

Figure S2
